# The Role of Neuroinflammation in the Comorbidity of Psychiatric Disorders and Internal Diseases

**DOI:** 10.3390/healthcare13070837

**Published:** 2025-04-07

**Authors:** Grecu Ramona Cătălina, Victor Gheorman, Veronica Gheorman, Mircea-Cătălin Forțofoiu

**Affiliations:** 1 University of Medicine and Pharmacy of Craiova, 200349 Craiova, Romania; grecu.ramonacatalina@yahoo.com; 2 Department of Psychiatry, University of Medicine and Pharmacy of Craiova, 200349 Craiova, Romania; 3 Department of Medical Semiology, University of Medicine and Pharmacy of Craiova, 200349 Craiova, Romania; catalin.fortofoiu@umfcv.ro

**Keywords:** neuroinflammation, psychiatric comorbidities, systemic inflammation, HPA axis dysregulation, IL-6 inhibitors, integrated care models

## Abstract

Psychiatric disorders and internal diseases frequently co-occur, posing significant challenges due to overlapping symptoms, shared pathophysiological mechanisms, and increased healthcare burdens. Neuroinflammation has emerged as a central mechanism linking these conditions, driven by systemic inflammation, hypothalamic–pituitary–adrenal (HPA) axis dysregulation, and autonomic nervous system (ANS) imbalance. This review synthesizes current evidence on the role of neuroinflammation in comorbid conditions such as depression, anxiety, cardiovascular disease, and diabetes mellitus, emphasizing bidirectional relationships and shared inflammatory pathways. This analysis identifies gaps in longitudinal studies, biomarker validation, and the integration of multidisciplinary care models. Emerging therapeutic approaches, including IL-6 inhibitors, vagus nerve stimulation, and behavioral interventions, show promise but remain underexplored in combined applications. Furthermore, disparities in research representation limit the generalizability of findings and highlight the need for inclusive clinical trials. Addressing these gaps through precision medicine, advanced biomarker monitoring technologies, and equitable healthcare strategies could transform the management of these complex comorbidities. By advancing our understanding of neuroinflammatory mechanisms and promoting integrated interventions, this review underscores the need for a collaborative, patient-centered approach to improve outcomes and reduce the global burden of psychiatric and internal disease comorbidities.

## 1. Introduction

The interplay between psychiatric disorders and internal diseases represents a critical challenge in contemporary healthcare. These comorbidities are not only prevalent but also profoundly impactful, contributing to increased morbidity, mortality, and healthcare costs worldwide [1]. Studies have shown that individuals with co-occurring psychiatric and chronic medical conditions face a twofold increase in all-cause mortality and a fivefold increase in suicide mortality, underscoring the urgent need for integrated care approaches [2]. Furthermore, severe mental disorders such as schizophrenia, major depression, and bipolar disorder are associated with a 10–20 years reduction in life expectancy, primarily due to cardiovascular diseases, diabetes, and infections [3]. Additionally, individuals with mental health comorbidities have a 24% higher risk of mortality, further illustrating the substantial impact of these conditions on global health [4].

Neuroinflammation is increasingly recognized as a central mechanism underlying a wide range of these comorbidities, with significant implications for global healthcare systems [5]. This recognition is particularly relevant in the post-pandemic era, where the burden of mental health conditions and systemic inflammation has intensified, highlighting the urgent need for integrated, multidisciplinary approaches [6]. By understanding these neuroinflammatory pathways, healthcare systems can develop precision medicine strategies to reduce the global burden of comorbidities. The economic impact of these comorbidities extends beyond healthcare costs, affecting workforce productivity and social support systems, further emphasizing the need for immediate action.

**Figure 1:**
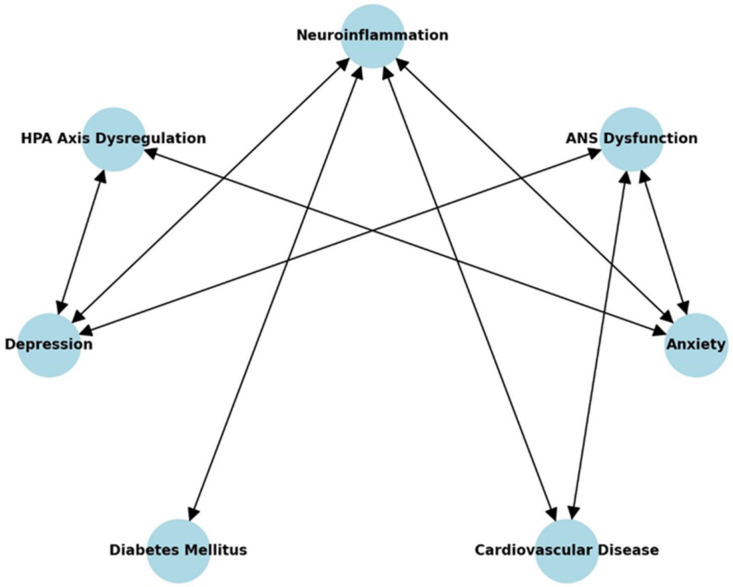
Pathophysiological mechanisms linking neuroinflammation, psychiatric disorders, and internal diseases.

This table provides supporting references for the pathways illustrated in Figure 1, showing the mechanistic links between neuroinflammation, psychiatric disorders, and internal diseases (Table 1).

### 1.1. Background

Comorbidities between psychiatric disorders and internal diseases represent a significant global health burden, affecting millions of individuals and imposing a substantial economic strain on healthcare systems [13]. Depression affects approximately 5% of adults worldwide [14] and is strongly associated with cardiovascular disease (CVD), which affects around 32% of the global population and remains the leading cause of mortality [15]. It is estimated that approximately 20–30% of individuals with CVD also experience clinical depression, a condition that nearly doubles the risk of adverse cardiovascular outcomes, including mortality [16,17]. Beyond depression and CVD, anxiety disorders and diabetes mellitus also share complex, interwoven relationships with systemic inflammation, further complicating clinical care [18,19]. Studies have shown that individuals with diabetes are more likely to experience these mental health conditions compared to the general population. For instance, a study reported that 42.3% of type 2 diabetes mellitus patients had depression symptoms, and 40.4% had anxiety symptoms [20]. Similarly, another study found that 27.5% of diabetic patients experienced anxiety, and 19.8% experienced depression, rates significantly higher than those in the general population [21]. A meta-analysis also estimated the prevalence of depression among diabetics to be twice that of the general population [22].

The economic implications of these conditions are profound, with increased healthcare utilization and reduced workforce productivity further exacerbating societal burdens. For instance, the costs associated with managing comorbid depression and CVD are estimated to be significantly higher than treating either condition alone, placing a disproportionate burden on healthcare systems and families [23].

The burden of these comorbidities is amplified by overlapping symptomatology, delayed diagnosis and fragmented care. For example, individuals with depression often experience fatigue, poor appetite, and sleep disturbances, symptoms that overlap with those of cardiovascular and metabolic diseases [23,24]. These overlapping symptoms not only challenge diagnostic accuracy but also contribute to poorer treatment adherence, thereby worsening patient outcomes. For instance, depression has been identified as an independent risk factor for poor prognosis in patients with heart failure, leading to increased hospitalizations and higher mortality rates [25]. Addressing these multifaceted challenges requires targeted interventions and policies to mitigate their impact on both individual and societal levels.

### 1.2. Role of Neuroinflammation in Psychiatric Disorders and Internal Disease

Neuroinflammation has emerged as a central mechanism linking psychiatric disorders and internal diseases [26]. Systemic inflammation, characterized by elevated levels of cytokines such as interleukin-6 (IL-6), tumor necrosis factor-alpha (TNF-α), and C-reactive protein (CRP), plays a pivotal role in bridging these conditions [27]. Elevated CRP levels, for instance, have been consistently associated with both major depressive disorder and an increased risk of myocardial infarction [28]. Chronic activation of the hypothalamic–pituitary–adrenal (HPA) axis and dysregulation of the autonomic nervous system further perpetuate systemic and neuroinflammation, creating a vicious cycle of disease progression [9]. These inflammatory pathways not only clarify the mechanisms underlying these comorbidities but also provide actionable targets for therapeutic interventions.

In depression and CVD, inflammatory pathways contribute to endothelial dysfunction, atherosclerosis, and dysregulated neurotransmitter systems [29]. This interplay exacerbates disease severity, delays recovery, and worsens quality of life. Similarly, in anxiety disorders, chronic inflammation exacerbates stress responses and contributes to hyperactivation of the amygdala and prefrontal cortex [8]. In diabetes mellitus, systemic inflammation is both a cause and a consequence of insulin resistance and glucose dysregulation, creating a bidirectional relationship that increases the risk of mental health disorders [12]. These shared pathophysiological underpinnings underscore the importance of studying neuroinflammation as a unifying mechanism in these comorbidities.

**Figure 2:**
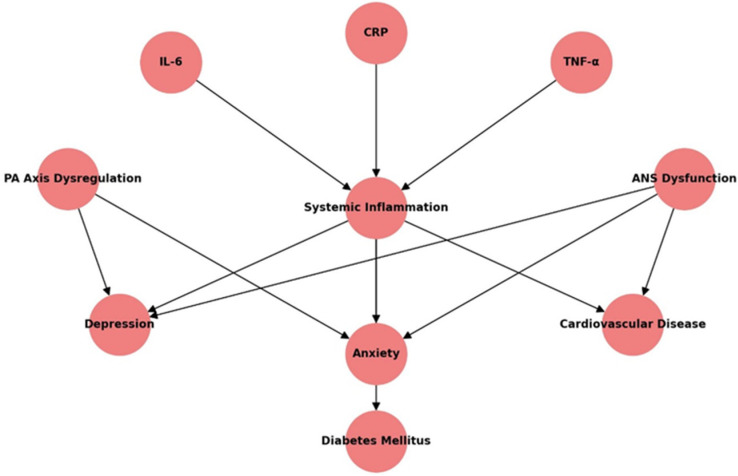
Systemic inflammation and its role in comorbidities.

This table provides supporting references for the pathways illustrated in Figure 2, showing the mechanistic links between systemic inflammation and its role in comorbidities(Table 2).

### 1.3. Objective

This narrative review aims to analyze the role of neuroinflammation in the intersection of psychiatric disorders and internal diseases, focusing primarily on depression and cardiovascular disease, with additional exploration of anxiety and diabetes mellitus. This review seeks to synthesize current evidence on shared mechanisms, identify diagnostic and therapeutic challenges, and highlight gaps in the literature to inform future research and clinical care. By identifying actionable gaps in the current literature, this review seeks to guide the development of integrated therapeutic and diagnostic approaches that address both the psychological and physiological dimensions of comorbidities.

## 2. Depression and Cardiovascular Disease

### 2.1. Shared Pathophysiological Mechanisms

#### 2.1.1. Systemic Inflammation and Endothelial Dysfunction

Systemic inflammation is a well-established mechanism linking depression and cardiovascular disease (CVD). Pro-inflammatory cytokines, including interleukin-6 (IL-6), tumor necrosis factor-alpha (TNF-α), and C-reactive protein (CRP), are significantly elevated in both conditions [11]. In patients with major depressive disorder (MDD), elevated CRP levels are observed in approximately 30–40% of cases and have been associated with a twofold increased risk of myocardial infarction and other adverse cardiovascular events [10,40]. Similarly, elevated IL-6 levels contribute to vascular injury by promoting oxidative stress, endothelial dysfunction, and the development of atherosclerosis [41].

Endothelial dysfunction, characterized by reduced nitric oxide bioavailability and impaired vasodilation, creates a pro-atherogenic environment that accelerates plaque formation and instability [42]. These inflammatory processes not only compromise vascular integrity but also directly interfere with mood regulation through the activation of the kynurenine pathway, reducing serotonin synthesis. The interplay between systemic inflammation and vascular dysfunction thus perpetuates a vicious cycle, exacerbating both cardiovascular and psychiatric outcomes.

#### 2.1.2. HPA Axis Dysregulation

The hypothalamic–pituitary–adrenal (HPA) axis is a central regulator of the stress response, and its dysregulation is a hallmark of both depression and CVD [43]. Chronic stress, a core component of MDD, leads to sustained activation of the HPA axis, resulting in hypercortisolemia [7]. Elevated cortisol levels contribute to systemic inflammation by upregulating cytokine production, including IL-6 and TNF-α, and suppressing anti-inflammatory pathways.

Prolonged HPA axis activation has deleterious effects on cardiovascular health. Hypercortisolemia is associated with hypertension, visceral adiposity, and insulin resistance, all of which are major risk factors for CVD [44]. Additionally, persistent cortisol exposure induces hippocampal atrophy and prefrontal cortex dysfunction, impairing emotional regulation and perpetuating depressive symptoms [45]. Longitudinal studies have demonstrated that individuals with chronic HPA axis dysregulation exhibit a significantly higher risk of cardiovascular events, highlighting the bidirectional nature of this relationship [46,47].

#### 2.1.3. Autonomic Nervous System Dysregulation

The autonomic nervous system (ANS) plays a pivotal role in maintaining homeostasis, and its dysregulation is increasingly recognized as a shared mechanism in depression and CVD [48]. Depression is characterized by heightened sympathetic activity and reduced parasympathetic tone, resulting in increased heart rate, reduced heart rate variability (HRV), and elevated vascular resistance [49]. These changes are associated with a higher incidence of adverse cardiovascular events, including sudden cardiac death.

Reduced HRV, a robust marker of autonomic dysfunction, has been identified as a predictor of both depressive severity and cardiovascular mortality [50]. Mechanistically, ANS dysregulation exacerbates systemic inflammation by increasing sympathetic-driven cytokine production and impairing vagally mediated anti-inflammatory reflexes [51]. This autonomic imbalance not only amplifies inflammation but also contributes to emotional dysregulation, creating a self-reinforcing cycle that worsens both conditions.

#### 2.1.4. Clinical Implications

The shared pathophysiological mechanisms of systemic inflammation, HPA axis dysregulation, and ANS imbalance underscore the need for integrated diagnostic and therapeutic approaches. Biomarkers such as CRP, IL-6, and HRV could serve as valuable tools for identifying high-risk patients and monitoring treatment efficacy. Emerging therapies targeting these pathways, including IL-6 inhibitors, vagus nerve stimulation, and stress reduction interventions, hold promise for improving both cardiovascular and psychiatric outcomes.

Future research should prioritize longitudinal studies to elucidate causal relationships and explore the efficacy of combined therapeutic strategies, particularly in patients with established comorbidities. Addressing these shared mechanisms holistically may not only improve individual outcomes but also reduce the overall burden of these interconnected conditions.

### 2.2. Bidirectional Relationship

The relationship between depression and cardiovascular disease (CVD) is inherently bidirectional, with each condition exacerbating the other through interlinked behavioral, physiological and inflammatory pathways [52]. Understanding this interplay is essential for developing integrated therapeutic strategies and addressing the shared burden of these conditions.

#### 2.2.1. Depression’s Effect on Cardiovascular Disease

Depression is a significant independent risk factor for the development and progression of CVD [53]. This impact is mediated by multiple mechanisms:

##### Poor Treatment Adherence and Lifestyle Behaviors

Depression impairs motivation and self-regulation, leading to reduced adherence to prescribed cardiovascular therapies such as antihypertensives and lipid-lowering agents [54]. Patients with depression are up to 50% less likely to participate in cardiac rehabilitation programs, contributing to increased hospital readmissions and mortality [55]. Moreover, unhealthy behaviors, including physical inactivity, smoking, and excessive alcohol consumption further amplify cardiovascular risk [55].

##### Inflammatory Pathways

Systemic inflammation is central to the pathophysiology of depression’s impact on CVD [56]. Elevated levels of C-reactive protein (CRP), interleukin-6 (IL-6), and tumor necrosis factor-alpha (TNF-α) in depressed individuals promote vascular injury and atherogenesis [11]. These inflammatory markers also exacerbate endothelial dysfunction by impairing nitric oxide synthesis, increasing oxidative stress and fostering arterial plaque formation [57].

##### HPA Axis Activation

The activation of the hypothalamic–pituitary–adrenal (HPA) axis in depression results in sustained hypercortisolemia, which drives insulin resistance, central adiposity, and hypertension [58]. Together, these metabolic changes significantly increase cardiovascular risk.

#### 2.2.2. Cardiovascular Disease’s Effect on Depression

Cardiovascular disease, in turn, acts as a significant risk factor for the development of depression, particularly in patients with chronic or advanced cardiac conditions [59].

##### Chronic Illness Stress

Living with a chronic cardiac condition imposes psychological and emotional stress, characterized by fear of recurrent events, reduced quality of life, and loss of autonomy. Studies estimate that approximately 15–20% of patients with CVD develop major depressive disorder within the first year following a cardiac event, with another 20–30% experiencing subclinical depressive symptoms [60,61].

##### Systemic and Neuroinflammation

Systemic inflammation associated with CVD exacerbates neuroinflammation, which disrupts mood regulation [62]. Elevated levels of IL-1β and TNF-α have been shown to impair serotonin and dopamine pathways, contributing to depressive symptoms [63]. This feedback loop between systemic inflammation and neuroinflammation perpetuates both conditions.

##### Cerebral Perfusion Deficits

Advanced CVD can lead to reduced cardiac output, compromising cerebral perfusion [64]. Hypoxia in critical brain regions, such as the hippocampus and prefrontal cortex, disrupts neural circuits involved in emotional regulation, further contributing to depressive states [65].

#### 2.2.3. Integrated Clinical Implications

The bidirectional relationship between depression and CVD necessitates a paradigm shift toward integrated care approaches that address both conditions concurrently. Key strategies include:

Routine Screening: Implement systematic screening for depressive symptoms in patients with CVD and cardiovascular risk factors in individuals with depression. Early identification allows for timely intervention and improved outcomes.

The frequency of screening for depressive symptoms in patients with CVD and for cardiovascular risk factors in individuals with depression should follow established clinical guidelines:

Depression Screening in CVD Patients: The American Heart Association (AHA) recommends annual screening for depression in patients with coronary artery disease (CAD) and other cardiovascular conditions, with additional assessments as needed for high-risk individuals [66].

Cardiovascular Risk Screening in Depression Patients: Given the increased CVD risk in individuals with depression, the European Society of Cardiology (ESC) suggests screening for hypertension, dyslipidemia, and diabetes at least annually in patients with major depressive disorder (MDD) [67]. Patients with severe or recurrent depression may benefit from more frequent cardiovascular assessments, particularly if they have additional risk factors such as obesity or smoking.

These structured, evidence-based screening approaches ensure that both conditions are adequately monitored and managed, improving patient outcomes and reducing long-term healthcare burdens.

Behavioral Interventions: Structured physical activity and cognitive-behavioral therapy have demonstrated efficacy in reducing depressive symptoms and improving cardiovascular health [68].

Pharmacological Therapies: Emerging treatments targeting shared mechanisms, such as IL-6 inhibitors and vagus nerve stimulation, offer potential for mitigating systemic inflammation and autonomic dysregulation [69].

#### 2.2.4. Future Directions

While substantial progress has been made in understanding the interplay between depression and CVD, critical gaps remain:

Causal Pathways: Longitudinal studies are needed to clarify causality and establish temporal relationships between depressive and cardiovascular outcomes.

Biomarker Development: Novel biomarkers, such as microRNAs (miRNAs) and advanced imaging techniques, could enhance risk prediction and treatment monitoring. miRNAs are small, non-coding RNA molecules that regulate gene expression and have emerged as promising biomarkers for both depression and CVD due to their stability in body fluids and specific expression patterns. In depression, miRNAs influence neuroplasticity and neurogenesis, processes implicated in mood regulation. Studies have identified specific miRNAs, such as miR-16, miR-182, miR-223, and miR-451, as potential biomarkers for major depressive disorder (MDD) [70]. In cardiovascular diseases, circulating miRNAs have been associated with various cardiovascular pathologies, including heart failure and myocardial infarction, highlighting their prognostic value [71]. Ongoing research continues to refine the role of miRNAs in risk stratification, aiming to integrate them into clinical practice for earlier detection and targeted therapeutic interventions [72].

Personalized Interventions: Research should focus on individualized therapeutic strategies that integrate behavioral, pharmacological, and biological approaches tailored to patient needs.

By addressing these gaps and leveraging shared pathophysiological mechanisms, future research can pave the way for more effective, integrated, and patient-centered interventions to mitigate the dual burden of depression and CVD.

### 2.3. Diagnostic and Therapeutic Challenges

The interplay between depression and cardiovascular disease (CVD) presents unique diagnostic and therapeutic challenges due to overlapping symptoms, treatment dilemmas, and the need for innovative therapeutic approaches. Addressing these challenges is critical for improving patient outcomes and reducing the burden of these comorbid conditions.

#### 2.3.1. Overlapping Symptoms

One of the most significant diagnostic challenges in managing comorbid depression and CVD lies in the overlap of symptoms such as fatigue, sleep disturbances, and reduced physical activity [24]. These nonspecific symptoms are hallmarks of both conditions, making it difficult to distinguish whether they stem from underlying cardiac pathology, depression, or both. For example:

Fatigue: While chronic fatigue is a cardinal symptom of depression, it is also a key feature of heart failure and other cardiac conditions [73].

Sleep Disturbances: Insomnia or hypersomnia is prevalent in major depressive disorder (MDD), but obstructive sleep apnea and other sleep disorders are common in patients with CVD [74,75].

The lack of specificity in these symptoms often delays accurate diagnosis and appropriate treatment, resulting in suboptimal management. Emerging biomarkers, such as CRP, IL-6, and heart rate variability (HRV), could aid in distinguishing between these conditions, but further validation is needed to integrate them into routine clinical practice.

#### 2.3.2. Treatment Dilemmas

Treating depression in patients with CVD poses several challenges due to the safety and efficacy concerns associated with antidepressants and other therapies.

1. Pharmacological Safety:

Selective serotonin reuptake inhibitors (SSRIs) are commonly prescribed for depression, but their use in cardiac patients requires caution [76]. While SSRIs such as sertraline and escitalopram are generally considered safe, some antidepressants can prolong QT intervals, increasing the risk of arrhythmias [77].

Tricyclic antidepressants (TCAs) are contraindicated in most cardiac patients due to their potential to exacerbate tachycardia, orthostatic hypotension, and conduction abnormalities [77,78].

2. Efficacy in Dual Pathologies:

Antidepressants often take weeks to demonstrate efficacy, during which depressive symptoms may continue to negatively impact cardiovascular outcomes.

The impact of antidepressant treatment on inflammatory markers remains inconsistent, raising questions about their ability to address shared underlying mechanisms.

3. Polypharmacy Risks:

Patients with CVD often take multiple medications for conditions such as hypertension, dyslipidemia, and diabetes [79]. Introducing antidepressants increases the risk of drug–drug interactions, complicating management [80].

These concerns highlight the need for comprehensive care strategies that integrate pharmacological, behavioral, and psychosocial approaches to address both conditions effectively.

4. Emerging Interventions

Recent advances in understanding the shared pathophysiological mechanisms of depression and CVD have paved the way for novel therapeutic approaches targeting systemic inflammation and autonomic dysregulation:

##### Anti-Inflammatory Therapies

IL-6 inhibitors (e.g., tocilizumab) have shown promise in reducing systemic inflammation and improving mood symptoms, particularly in patients with treatment-resistant depression [81,82]. Their dual action on inflammatory and cardiovascular pathways represents a potential breakthrough in managing these comorbidities.

Trials with TNF-α inhibitors and broader anti-inflammatory agents, such as statins have also demonstrated potential in mitigating depressive symptoms and reducing cardiovascular risk [83,84].

##### Vagus Nerve Stimulation (VNS)

VNS has emerged as a promising intervention that targets autonomic dysfunction and systemic inflammation. By enhancing parasympathetic activity, VNS may reduce both depressive symptoms and cardiovascular stress, with early trials showing encouraging results in patients with refractory depression [85,86].

##### Behavioral Interventions

Structured physical activity and mindfulness-based stress reduction have demonstrated efficacy in alleviating depressive symptoms and improving cardiovascular health [87]. These interventions also modulate inflammatory markers, providing a non-pharmacological approach to targeting shared mechanisms [88].

#### 2.3.3. Clinical Implications

The diagnostic and therapeutic challenges in managing depression and CVD highlight the importance of integrated care. Multidisciplinary teams that include cardiologists, psychiatrists, and behavioral health specialists are essential for implementing tailored interventions. Additionally, routine use of biomarkers and emerging technologies, such as wearable devices for monitoring HRV and inflammation, could improve diagnostic precision and therapeutic outcomes [89].

#### 2.3.4. Gaps and Future Directions

Refining Biomarkers: Further research is needed to validate biomarkers such as CRP, IL-6, and HRV for clinical use in differentiating depression from cardiac pathology.

Personalized Medicine: The development of personalized treatment protocols that account for individual inflammatory and autonomic profiles could optimize outcomes [90].

Long-Term Efficacy of Emerging Therapies: Rigorous trials are required to evaluate the safety and long-term efficacy of interventions like IL-6 inhibitors and VNS in managing these comorbidities.

## 3. Anxiety Disorders

### 3.1. Neuroinflammatory Mechanisms

#### 3.1.1. Immune Dysregulation in Anxiety

Anxiety disorders, among the most prevalent psychiatric conditions, are increasingly recognized as being influenced by immune dysregulation [91]. Chronic stress, a key component of anxiety, triggers the release of stress hormones such as cortisol, which subsequently dysregulates the immune system [92]. This dysregulation manifests as increased production of pro-inflammatory cytokines, including interleukin-1 beta (IL-1β), tumor necrosis factor-alpha (TNF-α), and interleukin-6 (IL-6) [93]. Elevated levels of these cytokines are consistently observed in individuals with anxiety disorders and have been directly correlated with symptom severity [94].

For instance, IL-6 has been implicated in exacerbating hypervigilance and somatic anxiety symptoms by modulating neural activity in the hypothalamus and amygdala [95]. Similarly, TNF-α influences the hypothalamic–pituitary–adrenal (HPA) axis, promoting dysregulation that exacerbates the physiological and psychological symptoms of anxiety [96]. Chronic inflammation also contributes to alterations in neurochemical systems, such as serotonin and gamma-aminobutyric acid (GABA), further amplifying anxiety severity [97].

#### 3.1.2. Brain–Immune Axis Dysfunction

The bidirectional communication between the immune system and the brain, termed the brain–immune axis, plays a critical role in the pathophysiology of anxiety disorders [98]. Neuroinflammation arising from systemic inflammation alters neural circuits involved in fear and stress regulation, including the amygdala, prefrontal cortex and hippocampus [99].

Pro-inflammatory cytokines, such as IL-1β, penetrate the blood–brain barrier and activate microglial cells, initiating a neuroinflammatory response [100]. This process disrupts the balance of excitatory and inhibitory neurotransmission, heightening amygdala reactivity while impairing the regulatory functions of the prefrontal cortex [101]. These changes exacerbate maladaptive fear responses, perpetuating the cycle of anxiety.

Furthermore, chronic inflammation-induced neural remodeling in the hippocampus contributes to impairments in stress adaptation and memory consolidation, both of which are critical in the pathogenesis of anxiety disorders [102]. The interplay between peripheral inflammation and central neuroinflammation highlights the importance of targeting the brain–immune axis for therapeutic interventions.

### 3.2. Comorbidities with Internal Diseases

#### 3.2.1. Hypertension and Gastrointestinal Disorders

Anxiety disorders significantly exacerbate the progression of internal diseases, such as hypertension and gastrointestinal (GI) disorders, through systemic inflammation and dysregulation of the autonomic nervous system (ANS) [103]. Chronic anxiety-induced stress perpetuates a physiological state of heightened sympathetic activity and suppressed parasympathetic tone, disrupting the homeostatic mechanisms of internal organs [104].

#### 3.2.2. Hypertension

Chronic anxiety has been consistently linked to the development and worsening of hypertension through its effects on systemic inflammation and ANS dysregulation [105]. Persistent activation of the hypothalamic–pituitary–adrenal (HPA) axis elevates cortisol levels, which in turn increases pro-inflammatory cytokines such as interleukin-6 (IL-6) and tumor necrosis factor-alpha (TNF-α) [106]. These inflammatory mediators promote endothelial dysfunction by reducing nitric oxide bioavailability, leading to impaired vasodilation and increased vascular resistance.

Moreover, heightened sympathetic nervous system activity in anxiety disorders contributes to sustained elevations in blood pressure by increasing heart rate, vascular tone, and renin–angiotensin–aldosterone system (RAAS) activation [107]. This chronic hypertensive state not only damages vascular integrity but also feeds back into the anxiety pathway by exacerbating neuroinflammation and disrupting stress-regulating neural circuits, such as the prefrontal cortex and amygdala [108].

#### 3.2.3. Gastrointestinal Disorders

The bidirectional communication between the brain and the gut, often referred to as the gut–brain axis, plays a pivotal role in the development and progression of GI disorders in individuals with chronic anxiety [98]. Pro-inflammatory cytokines such as IL-1β, IL-6, and TNF-α disrupt intestinal epithelial integrity, increasing gut permeability (“leaky gut”) and facilitating the translocation of endotoxins into systemic circulation [109]. This perpetuates systemic inflammation, which exacerbates both anxiety and GI symptoms.

In conditions such as irritable bowel syndrome (IBS) and inflammatory bowel disease (IBD), chronic anxiety has been shown to worsen abdominal pain, diarrhea, and other GI symptoms through its effects on the enteric nervous system [110]. Dysregulation of the ANS contributes to abnormal gut motility, while heightened amygdala reactivity amplifies the perception of visceral pain [111]. Furthermore, alterations in gut microbiota composition, driven by stress-induced changes in dietary and immune factors, have been implicated in the exacerbation of GI inflammation and anxiety symptoms [112].

#### 3.2.4. Clinical Implications

Understanding the role of anxiety in exacerbating internal diseases such as hypertension and GI disorders underscores the importance of integrated care approaches. Effective management requires addressing both the psychological and physiological components of these comorbidities. Strategies may include:

Pharmacological Interventions: Targeting systemic inflammation with anti-cytokine therapies (e.g., IL-6 inhibitors) or ANS dysregulation with beta-blockers and vagus nerve stimulation.

Behavioral Interventions: Implementing cognitive-behavioral therapy (CBT) and mindfulness-based stress reduction to mitigate anxiety’s impact on systemic and gut inflammation.

Lifestyle Modifications: Encouraging regular physical activity and dietary interventions to support cardiovascular health and gut microbiota balance.

### 3.3. Treatment Perspectives

Management of anxiety disorders, particularly when comorbid with internal diseases, requires an integrative approach combining established psychological and pharmacological interventions with emerging immunomodulatory strategies. Targeting both the mental and physiological components of anxiety is essential to improve outcomes and mitigate its impact on comorbid conditions such as hypertension and gastrointestinal (GI) disorders.

#### 3.3.1. Psychological and Pharmacological Interventions

##### Cognitive-Behavioral Therapy (CBT)

CBT is the gold standard for anxiety treatment, addressing maladaptive thought patterns and behaviors while equipping patients with coping strategies [113]. In comorbid anxiety and internal diseases, tailored CBT protocols can incorporate stress-reduction techniques to alleviate sympathetic overactivation and systemic inflammation. For instance, meta-analyses demonstrate that CBT not only reduces anxiety severity but also improves physiological markers such as heart rate variability (HRV), benefiting patients with hypertension or GI disorders [114].

##### Anxiolytic Medications

Pharmacological treatments, such as selective serotonin reuptake inhibitors (SSRIs) and serotonin–norepinephrine reuptake inhibitors (SNRIs), are effective in reducing anxiety symptoms and addressing comorbid stress-related disorders [115]. However, their inconsistent effects on inflammatory markers such as interleukin-6 (IL-6) necessitate adjunctive strategies. Benzodiazepines, while effective for short-term relief, are often avoided in chronic anxiety due to risks of dependency, cognitive impairment, and adverse interactions with treatments for internal diseases [116].

#### 3.3.2. Emerging Immunomodulatory Therapies

Recent advancements in understanding the role of inflammation in anxiety pathophysiology have led to the exploration of immunomodulatory agents as potential treatments.

##### Anti-Inflammatory Agents

Cytokine Inhibitors: Targeted therapies such as IL-6 inhibitors (e.g., tocilizumab) and TNF-α blockers have shown promise in reducing anxiety symptoms in patients with high inflammatory markers [81].

Non-Steroidal Anti-Inflammatory Drugs (NSAIDs): Certain NSAIDs, such as celecoxib, have shown anxiolytic effects by reducing peripheral inflammation and its downstream impact on neuroinflammatory processes [117]. While promising, their long-term safety in chronic anxiety management requires further study.

##### Vagus Nerve Stimulation (VNS)

VNS is an emerging therapy that directly targets autonomic dysfunction and systemic inflammation [86]. By enhancing parasympathetic activity, VNS reduces anxiety severity and improves physiological outcomes, including HRV and GI motility. Bonaz et al. reported that VNS not only alleviated anxiety but also improved gut health in patients with irritable bowel syndrome, highlighting its potential in addressing comorbid GI disorders [118].

#### 3.3.3. Integrated Approaches

An integrated care model combining psychological, pharmacological, and immunomodulatory interventions is likely to yield the most substantial benefits. For example:

CBT and Anti-Inflammatory Therapies: Addressing both cognitive distortions and systemic inflammation simultaneously can improve both mental and physical health outcomes.

VNS and Lifestyle Modifications: Combining VNS with dietary interventions targeting gut microbiota health can mitigate inflammation and alleviate GI symptoms while reducing anxiety.

#### 3.3.4. Future Directions

To advance treatment strategies for anxiety disorders with comorbid internal diseases, future research should prioritize the following:

Personalized Therapies: Developing treatment protocols based on biomarkers of inflammation (e.g., IL-6, CRP) and autonomic dysfunction (e.g., HRV).

Combination Strategies: Investigating the synergistic effects of immunomodulatory and psychological interventions, such as combining CBT with IL-6 inhibitors.

Long-Term Efficacy Studies: Evaluating the safety, tolerability, and effectiveness of cytokine inhibitors and VNS in chronic anxiety management.

## 4. Diabetes Mellitus

### 4.1. Inflammation and Insulin Resistance

Diabetes mellitus, a chronic metabolic disorder, is closely linked to systemic inflammation, which exacerbates both insulin resistance and neuroinflammation [119]. Pro-inflammatory cytokines such as interleukin-1 beta (IL-1β) and tumor necrosis factor-alpha (TNF-α) play pivotal roles in the pathogenesis of diabetes by directly impairing pancreatic beta-cell function and promoting systemic metabolic dysregulation [120].

#### 4.1.1. Role of IL-1β and TNF-α

##### Pancreatic Beta-Cell Dysfunction

IL-1β and TNF-α are central mediators of beta-cell apoptosis and dysfunction. Chronic exposure to elevated glucose levels (glucotoxicity) and free fatty acids (lipotoxicity) triggers an inflammatory response within the islets of Langerhans, leading to the production of IL-1β and TNF-α by resident immune cells [121].

IL-1β exerts deleterious effects by activating the nuclear factor-kappa B (NF-κB) pathway, which promotes oxidative stress and reduces insulin secretion. Studies have shown that IL-1β receptor antagonists, such as anakinra, significantly improve beta-cell function and glycemic control in patients with type 2 diabetes [122].

Similarly, TNF-α exacerbates insulin resistance by impairing insulin receptor signaling. It reduces the activity of insulin receptor substrate proteins and increases serine phosphorylation of the insulin receptor, disrupting glucose uptake in peripheral tissues. Elevated TNF-α levels have been correlated with higher HbA1c levels and increased risk of diabetes-related complications [123].

#### 4.1.2. Impact of Hyperglycemia on Neuroinflammation

##### Systemic Inflammation and Neural Effects

Chronic hyperglycemia is a key driver of systemic inflammation, which subsequently influences neuroinflammatory processes [124]. High glucose levels promote the generation of advanced glycation end-products (AGEs) and reactive oxygen species (ROS), both of which activate pro-inflammatory pathways [125]. This contributes to endothelial dysfunction and compromises the integrity of the blood–brain barrier (BBB), facilitating the entry of inflammatory cytokines into the central nervous system.

##### Neuroinflammatory Pathways

Inflammatory mediators such as IL-6 and TNF-α disrupt neural homeostasis by activating microglial cells, leading to the release of additional pro-inflammatory cytokines [27]. This creates a feedback loop that perpetuates neuroinflammation, impairing cognitive function and increasing the risk of depression in diabetic patients. Research by Kappelmann et al. demonstrated that elevated systemic inflammation markers, including CRP and IL-6, are significantly associated with both depression and impaired glycemic control, highlighting the bidirectional relationship between metabolic dysfunction and mental health [126].

##### Hypothalamic Dysfunction

Hyperglycemia-induced inflammation also disrupts hypothalamic function, a critical regulator of energy homeostasis and glucose metabolism [127]. Studies have identified an inflammatory response in hypothalamic neurons mediated by IL-1β and TNF-α, which impairs insulin signaling and exacerbates hyperglycemia, further linking metabolic dysregulation with neuroinflammatory processes [128].

#### 4.1.3. Clinical Implications

Understanding the roles of IL-1β, TNF-α, and hyperglycemia in the interplay between inflammation and insulin resistance opens new therapeutic avenues. Anti-inflammatory treatments targeting these pathways, such as IL-1 receptor antagonists and TNF-α inhibitors, have shown promise in improving glycemic control and reducing systemic inflammation. Moreover, lifestyle interventions aimed at reducing glucose levels and inflammatory load, such as structured exercise and dietary modifications, are critical components of diabetes management.

#### 4.1.4. Future Directions

Biomarker Development: Further research is needed to identify and validate inflammatory biomarkers (e.g., IL-1β, TNF-α) for predicting diabetes progression and treatment response.

Combination Therapies: Exploring the synergistic effects of anti-inflammatory agents with standard antidiabetic treatments.

Longitudinal Studies: Investigating the long-term impact of reducing neuroinflammation on glycemic control and mental health outcomes in diabetic populations.

### 4.2. Bidirectional Relationship with Depression

The relationship between diabetes mellitus and depression is inherently bidirectional, with each condition exacerbating the other through interconnected physiological, behavioral, and psychological pathways [12]. Chronic inflammation, metabolic dysfunction, and psychological stress are central to diabetes increasing the risk of depression, while depression worsens diabetes outcomes by impairing self-management and heightening systemic inflammation [129].

The interplay between inflammatory and metabolic pathways in diabetes has profound implications for brain function and neurotransmitter balance. Elevated levels of pro-inflammatory cytokines such as IL-1β, TNF-α, and IL-6 contribute to oxidative stress, mitochondrial dysfunction, and increased blood–brain barrier (BBB) permeability, which in turn disrupts neurotransmission [130]. These inflammatory mediators impair serotonin synthesis by depleting tryptophan availability, disrupt dopamine signaling, and alter the GABA/glutamate balance, leading to increased excitotoxicity and neuronal dysfunction. Specifically, activation of the indoleamine 2,3-dioxygenase (IDO) pathway by inflammatory cytokines leads to serotonin depletion, contributing to mood disorders [130]. In addition, chronic inflammation affects dopamine metabolism, reducing dopamine synthesis, packaging, and release, which has been linked to depressive symptoms such as anhedonia and psychomotor slowing [131]. This cascade of events is closely linked to cognitive deficits, mood disorders, and heightened susceptibility to psychiatric comorbidities in individuals with diabetes.

#### 4.2.1. Diabetes Increasing Depression Risk

##### Chronic Inflammation as a Driver of Depression

Systemic inflammation is a hallmark of diabetes and plays a pivotal role in the development of depression. Pro-inflammatory cytokines such as interleukin-6 (IL-6), tumor necrosis factor-alpha (TNF-α), and C-reactive protein (CRP) disrupt the blood–brain barrier, facilitating the entry of these cytokines into the central nervous system [132]. Once in the brain, these cytokines activate microglial cells, which release additional inflammatory mediators and impair neurogenesis in the hippocampus, a region critical for mood regulation.

##### Metabolic Dysfunction and Neurochemical Changes

Hyperglycemia and insulin resistance, common features of diabetes, disrupt neural homeostasis by promoting oxidative stress and advanced glycation end-product (AGE) accumulation [133]. These processes impair neurotransmitter systems, particularly serotonin and dopamine, both of which are central to mood regulation. A study by Kappelmann et al. found that individuals with elevated CRP levels, a marker of systemic inflammation, were at significantly increased risk of developing depressive symptoms, further linking metabolic dysfunction with mental health [126].

##### Psychological Stress and Burden of Disease

The psychosocial burden of managing diabetes, including frequent monitoring of blood glucose, dietary restrictions and fear of complications, contributes to chronic stress and the development of depressive symptoms. Anderson et al. have demonstrated that individuals with diabetes are approximately twice as likely to develop depression compared to those without diabetes, highlighting the critical role of psychological factors in managing this condition [134].

#### 4.2.2. Depression Worsening Diabetes Outcomes

##### Poor Self-Management

Depression significantly impairs the ability of individuals to adhere to diabetes management regimens, including medication adherence, regular physical activity, and dietary modifications. A meta-analysis by Gonzalez et al. (2008) demonstrated that depression is significantly associated with nonadherence to diabetes self-care behaviors, contributing to poor glycemic control and a higher risk of complications [135].

##### Heightened Systemic Inflammation

Depression exacerbates systemic inflammation, further impairing glucose metabolism and insulin sensitivity [136]. Elevated levels of IL-6 and TNF-α in individuals with depression contribute to insulin resistance and pancreatic beta-cell dysfunction, creating a vicious cycle of metabolic and psychological dysregulation [136]. Elevated systemic inflammation in individuals with comorbid depression and diabetes exacerbates metabolic dysfunction and significantly increases the risk of cardiovascular events, highlighting the interconnected nature of these conditions [137].

##### Autonomic Nervous System (ANS) Dysregulation

Depression-induced dysregulation of the ANS, characterized by increased sympathetic activity and reduced parasympathetic tone, worsens glycemic control by impairing insulin secretion and promoting hepatic glucose output [138]. Reduced heart rate variability (HRV), a marker of ANS dysfunction, has been consistently associated with poor diabetes outcomes in individuals with comorbid depression [139].

#### 4.2.3. Clinical Implications

Understanding the bidirectional relationship between diabetes and depression highlights the need for integrated care approaches. Routine screening for depressive symptoms in individuals with diabetes and metabolic risk assessments in those with depression should become standard practice. Combining psychosocial interventions such as cognitive-behavioral therapy (CBT) with pharmacological treatments targeting both inflammation and glycemic control offers a promising avenue for improving outcomes [140].

#### 4.2.4. Future Directions

Personalized Treatment Strategies: Development of tailored interventions that address individual inflammatory and metabolic profiles in patients with comorbid depression and diabetes.

Biomarker Research: Investigating the utility of CRP, IL-6 and HRV as predictive markers for identifying patients at high risk of poor outcomes due to this bidirectional relationship.

Longitudinal Studies: Assessing the long-term impact of integrative therapeutic approaches, including the combined use of anti-inflammatory agents and behavioral therapies.

### 4.3. Emerging Treatment Strategies

The bidirectional relationship between diabetes and depression necessitates innovative treatment strategies that target shared pathophysiological mechanisms, such as systemic inflammation and neuroinflammation. Recent advancements in anti-inflammatory therapies and integrated care models offer promising approaches to improving both metabolic and mental health outcomes [141].

#### 4.3.1. Anti-Inflammatory Therapies

##### IL-1 Antagonists and Beta-Cell Preservation

Interleukin-1 beta (IL-1β) plays a critical role in both insulin resistance and pancreatic beta-cell dysfunction [142]. Clinical trials have demonstrated that IL-1 receptor antagonists, such as anakinra, significantly improve glycemic control and reduce systemic inflammation in patients with type 2 diabetes. Larsen et al. reported that anakinra not only improved HbA1c levels but also preserved beta-cell function, reducing the progression of diabetes [122].

##### Dual Benefits for Mental Health Outcomes

IL-1 antagonists have shown potential in reducing systemic and neuroinflammation, which are key drivers of depressive symptoms in some individuals. For example, studies have demonstrated that targeting inflammatory pathways, including IL-1, may improve mood symptoms in individuals with elevated inflammatory markers [143,144]. This suggests that IL-1 antagonists could serve as a promising therapy for individuals with comorbid diabetes and depression by addressing the shared inflammatory mechanisms underlying these conditions.

##### TNF-α Inhibitors and Insulin Sensitivity

Tumor necrosis factor-alpha (TNF-α) inhibitors, such as etanercept, have been studied for their role in reducing insulin resistance and improving inflammatory profiles [145]. Studies suggest that these agents can mitigate neuroinflammatory pathways associated with depression, further supporting their use in addressing comorbid conditions [145,146]. However, more research is needed to evaluate their long-term efficacy and safety in diabetic populations.

#### 4.3.2. Models of Integrated Care

##### Multidisciplinary Approaches

Integrated care models that bring together endocrinologists, psychiatrists, primary care physicians and behavioral health specialists are essential for addressing the complex interplay between diabetes and depression. These models focus on holistic care by targeting both physiological and psychological aspects of the diseases.

##### Collaborative Interventions

Diabetes Self-Management Education (DSME): Programs that combine education on glycemic control with psychological support have been shown to improve both HbA1c levels and depression severity [147].

Psychological Interventions in Clinical Settings: Embedding cognitive-behavioral therapy (CBT) within diabetes care clinics allows for early identification and management of depressive symptoms, reducing their impact on glycemic outcomes.

##### Technology-Driven Integration

The use of telemedicine and digital platforms to coordinate care has become increasingly important in integrated models [148]. Mobile applications and wearable devices can monitor both glycemic control and psychological symptoms, enabling real-time interventions. For example, digital CBT platforms have demonstrated efficacy in reducing depressive symptoms, particularly in individuals with chronic illnesses like diabetes [149].

##### Clinical Implications

Emerging therapies that address inflammation and integrated care models that bridge physical and mental health represent a paradigm shift in managing comorbid diabetes and depression. The implementation of these strategies into routine care could improve outcomes by targeting shared mechanisms and fostering patient-centered, multidisciplinary approaches.

##### Future Directions

Expansion of Anti-Inflammatory Trials: Conducting large-scale, longitudinal studies to evaluate the efficacy of IL-1 and TNF-α inhibitors in improving both metabolic and mental health outcomes.

Refinement of Integrated Care Models: Further research into cost-effective and scalable integrated care frameworks, particularly in underserved populations, is needed.

Technological Advancements: The development of advanced digital tools that integrate biomarker monitoring (e.g., CRP, IL-6) with psychological assessments could revolutionize care delivery.

## 5. Models of Integrated Care

The intricate interplay between psychiatric disorders and internal diseases, underpinned by shared mechanisms such as neuroinflammation, underscores the need for integrated care models that simultaneously address psychological and physiological dimensions. Multidisciplinary approaches are essential for optimizing outcomes, as they foster collaboration among specialists and enable the implementation of comprehensive, patient-centered treatment strategies.

### 5.1. Multidisciplinary Approaches

#### 5.1.1. Collaborative Models in Depression and Cardiovascular Disease

The bidirectional relationship between depression and cardiovascular disease (CVD) underscores the need for psychiatry–cardiology integration in clinical care [150]. These collaborative models aim to address systemic inflammation, autonomic nervous system dysregulation, and behavioral risk factors that exacerbate both conditions.

##### Case Examples of Integration

- Co-located Care Models

In co-located clinics, psychiatrists and cardiologists work together to screen and manage patients with comorbid depression and CVD. For example, programs embedding mental health professionals in cardiac rehabilitation units have demonstrated improvements in both mood symptoms and cardiac outcomes [151]. Patients receiving integrated care report higher adherence to cardiovascular medications and exercise regimens, leading to reduced hospitalization rates and improved quality of life [151].

- Integrated Screening Protocols

Routine use of depression screening tools, such as the Patient Health Questionnaire-9 (PHQ-9), in cardiology practices facilitates early identification of depressive symptoms [152]. Simultaneously, cardiovascular risk assessments in psychiatric settings enable the proactive management of conditions like hypertension and dyslipidemia, reducing long-term morbidity [153].

##### Shared Benefits

Collaborative models address not only the neuroinflammatory mechanisms underlying both conditions but also improve treatment adherence by reducing fragmentation in care [154]. These approaches are particularly effective in targeting inflammatory pathways through combined behavioral, pharmacological, and lifestyle interventions.

#### 5.1.2. Comprehensive Care for Anxiety and Diabetes

Anxiety disorders and diabetes mellitus are interconnected through systemic inflammation, autonomic dysfunction, and the psychosocial burden of managing chronic diseases [155]. Multidisciplinary care models that combine behavioral, medical, and nutritional interventions are crucial in managing these complex comorbidities [156].

##### Behavioral Interventions

Cognitive-behavioral therapy (CBT) tailored for individuals with diabetes and anxiety has shown to reduce anxiety symptoms while improving glycemic control [140,157]. By addressing maladaptive thought patterns and stress-related behaviors, CBT mitigates the impact of anxiety on blood glucose levels and systemic inflammation.

##### Medical Interventions

Incorporating psychiatric care into diabetes management allows for the optimization of pharmacological therapies. For instance:

SSRIs: These are used not only for anxiety reduction but also for their potential anti-inflammatory effects, addressing both neuroinflammation and systemic inflammation [158].

Anti-inflammatory Agents: Emerging evidence supports the use of IL-6 inhibitors and NSAIDs to target inflammation-related pathways that exacerbate both anxiety and metabolic dysfunction [159].

##### Nutritional Support

Dietary interventions focusing on anti-inflammatory foods, such as omega-3 fatty acids and antioxidants, have been shown to benefit both mental health and glycemic control [160]. Collaboration with dietitians in comprehensive care models ensures that patients receive tailored nutritional guidance that supports overall health.

##### Examples of Comprehensive Care

Integrated Behavioral and Nutritional Clinics: Clinics providing combined CBT, mindfulness-based stress reduction, and personalized dietary counseling have reported significant improvements in HbA1c levels, anxiety severity, and overall patient satisfaction [161,162].

Remote Monitoring Tools: The integration of telemedicine platforms allows healthcare teams to track both glycemic and psychological parameters, facilitating timely interventions and fostering better engagement in self-management [163].

#### 5.1.3. Clinical Implications

Integrated care models targeting the shared mechanisms of neuroinflammation in psychiatric disorders and internal diseases are essential for improving outcomes. By addressing both psychological and physiological aspects, these approaches reduce healthcare fragmentation and enhance patient-centered care.

#### 5.1.4. Future Directions

Scaling Multidisciplinary Models: Research should focus on scaling integrated care models to diverse healthcare settings, particularly in underserved populations.

Technology-Enhanced Care: Advanced wearable devices and telemedicine platforms should be further developed to enable real-time monitoring and facilitate multidisciplinary collaboration.

Validation of Protocols: Standardized protocols for psychiatry–cardiology and psychiatry–endocrinology integration need to be established and validated through randomized controlled trials.

### 5.2. Emerging Technologies

The integration of advanced technologies into healthcare systems has revolutionized the management of comorbid psychiatric and internal diseases [164]. For conditions such as depression, anxiety, cardiovascular disease (CVD), and diabetes mellitus, emerging technologies offer innovative tools for monitoring neuroinflammation and improving treatment precision [165]. These advancements align with the growing demand for personalized, data-driven care, addressing both physiological and psychological aspects of these comorbidities.

#### 5.2.1. Telemedicine for Monitoring Comorbidities

Telemedicine has become an indispensable tool for managing comorbid psychiatric and internal diseases by enabling remote monitoring of inflammation and psychological symptoms [163]. It bridges the gap between patients and multidisciplinary teams, fostering continuous and proactive care.

##### Remote Monitoring of Inflammation and Mental Health

Telemedicine platforms, combined with wearable technologies, enable real-time monitoring of biomarkers such as heart rate variability (HRV), providing insights into autonomic dysfunction and systemic health. While continuous monitoring of inflammatory markers like C-reactive protein (CRP) and interleukin-6 (IL-6) remains in the developmental stage, ongoing research suggests promising applications for integrating these tools into future telemedicine practices, particularly for managing chronic inflammatory conditions [166,167,168]. For instance:

Inflammatory Monitoring: Regular CRP and IL-6 assessments via home-based diagnostic devices help in identifying early signs of disease exacerbation, enabling timely interventions [169,170].

Psychological Symptom Assessment: Digital tools such as mobile apps using validated questionnaires (e.g., PHQ-9 for depression, GAD-7 for anxiety) provide continuous feedback on mental health status [171]. This ensures that psychological symptoms are not overlooked in patients with chronic internal diseases.

##### Examples of Clinical Application

Integrated Telemedicine Platforms: Platforms combining physiological data (e.g., glucose levels, blood pressure) with psychological assessments enhance care coordination for patients with comorbid depression and diabetes.

Virtual Consultations: Telepsychiatry services integrated with cardiology and endocrinology care allow real-time adjustments in therapy based on patient-reported outcomes and biomarker trends.

##### Impact on Outcomes

Studies have demonstrated that telemedicine reduces hospitalization rates, improves treatment adherence, and enhances patient satisfaction by providing continuous, integrated care. While its ability to address neuroinflammatory pathways directly is still under investigation, telemedicine’s capacity to enable timely interventions and close monitoring plays a crucial role in managing complex comorbidities [163,172,173].

#### 5.2.2. Wearable Devices

Wearable devices have emerged as powerful tools for monitoring physiological and psychological parameters, providing actionable insights into the interplay between neuroinflammation, psychiatric disorders, and internal diseases [174].

##### Tracking Heart Rate Variability (HRV)

HRV is a robust marker of autonomic nervous system (ANS) activity and systemic inflammation. Wearable devices capable of continuously measuring HRV can detect early signs of autonomic dysfunction, which is a shared mechanism in conditions like depression, anxiety, and CVD [175]. For instance:

Low HRV: Indicates heightened sympathetic activity and reduced parasympathetic tone, correlating with worsened depressive symptoms and increased cardiovascular risk.

Monitoring Progress: Improvements in HRV during treatment (e.g., through vagus nerve stimulation or structured exercise) can serve as an indicator of therapeutic efficacy.

##### Glucose Monitoring and Metabolic Health

Continuous glucose monitors (CGMs) provide real-time data on glycemic fluctuations, enabling better management of diabetes and its neuroinflammatory consequences. These devices are particularly valuable in individuals with comorbid depression, where glycemic control is often compromised due to poor self-management [176,177].

##### Inflammatory Marker Detection

Emerging wearable technologies are being developed to non-invasively measure inflammatory biomarkers such as CRP and IL-6. By integrating these capabilities, wearable devices could transform the management of conditions where systemic inflammation is central, offering real-time feedback for clinical decision making [170,178].

##### Examples of Wearable Applications

Smartwatches with HRV Monitoring: Devices like Fitbit and Apple Watch have integrated HRV tracking capabilities, providing users and clinicians with insights into stress levels and autonomic function [179].

Hybrid Devices for Inflammatory Tracking: Experimental wearables capable of analyzing sweat or interstitial fluid for inflammatory markers are currently under development, holding promise for managing inflammation-driven diseases [180].

##### Clinical Benefits

Wearable devices empower patients by promoting self-monitoring and real-time feedback. They facilitate early detection of disease exacerbations, improve adherence to treatment plans, and enhance patient engagement in managing their health.

#### 5.2.3. Clinical Implications

The integration of telemedicine and wearable devices into care models addressing psychiatric and internal diseases has the potential to revolutionize management by providing real-time, personalized insights. These technologies bridge gaps in traditional care, enabling continuous monitoring and early intervention, particularly in targeting shared mechanisms such as neuroinflammation and autonomic dysregulation.

#### 5.2.4. Future Directions

Technological Advancement: Further development of non-invasive devices capable of detecting inflammatory markers (e.g., CRP, IL-6) is essential for enhancing disease monitoring.

Integration into Multidisciplinary Care: Incorporating telemedicine and wearable data into collaborative care models will improve care coordination and outcomes.

Validation in Large-Scale Trials: Rigorous testing of these technologies in diverse patient populations is needed to establish their efficacy and cost-effectiveness in managing comorbid psychiatric and internal diseases.

## 6. Discussion

The findings of this review underscore neuroinflammation as a central mechanism linking psychiatric disorders and internal diseases. While existing evidence highlights critical pathophysiological interactions, significant gaps remain in our understanding of causality, biomarker validation, and integrated treatment approaches. Addressing these challenges is essential for developing targeted interventions that bridge psychiatry, internal medicine, and inflammatory research.

### 6.1. Current Gaps in Research and Literature

1. Longitudinal Evidence and Causal Pathways

Existing studies are predominantly cross-sectional, limiting their ability to establish whether systemic inflammation is a precursor to psychiatric and internal comorbidities or a consequence of disease progression. It remains unclear whether neuroinflammation triggers the simultaneous onset of depression and cardiovascular disease or whether these conditions exacerbate each other over time [69].

Future Directions: Longitudinal cohort studies are imperative to map the temporal evolution of these comorbidities. These studies should integrate inflammatory biomarkers (e.g., IL-6, CRP) with clinical and behavioral data to identify early predictors of disease progression and inform timely, targeted interventions [181].

2. Biomarker Validation and Clinical Application

Although biomarkers such as IL-6, CRP, and HRV have been extensively linked to both psychiatric and internal diseases, their integration into routine clinical practice remains limited due to insufficient specificity and validation. For instance, IL-6 is consistently elevated in both depression and cardiovascular disease, yet its predictive value for dual comorbidities is not well established [182]. Similarly, while CRP is an accessible and cost-effective inflammatory marker, its diagnostic precision in differentiating primary psychiatric disorders from inflammation-driven internal diseases requires further validation across diverse populations [183].

Future Directions: Research should prioritize the development of validated, non-invasive diagnostic tools that operationalize these biomarkers for real-world clinical application. IL-6 and CRP hold potential as dual-purpose biomarkers, but their specificity and predictive accuracy require rigorous multicenter validation [184].

Technological innovations, such as wearable biosensors, offer promising solutions to bridge this gap. Biosensors capable of detecting inflammatory markers in real time via sweat or interstitial fluid analysis are in development, providing dynamic feedback on systemic inflammation [185]. A proposed study design could assess the efficacy of integrating biomarker monitoring with targeted interventions (e.g., IL-6 inhibitors, structured behavioral therapies) through a randomized controlled trial. Outcome measures should include reductions in systemic inflammation (CRP, IL-6), psychiatric symptom improvement (PHQ-9, GAD-7), and cost-effectiveness [186].

3. Integrated Therapeutic Interventions

Despite growing evidence of shared inflammatory and neurobiological mechanisms, most current treatments remain compartmentalized, addressing either psychiatric or internal diseases independently. Integrated anti-inflammatory and neuropsychiatric approaches remain largely unexplored.

Future Directions: Combining biological, pharmacological, and behavioral therapies offers a promising avenue for intervention.

- Vagus nerve stimulation (VNS): Clinical trials have shown that VNS reduces depressive symptoms in treatment-resistant depression while modulating systemic inflammation, with documented reductions in TNF-α and IL-6 levels [69].
- Mindfulness-Based Stress Reduction (MBSR): Studies have linked MBSR to reductions in systemic inflammation (CRP) and improvements in psychological resilience, yet its integration into multimodal treatment frameworks remains underexplored [181].
- Immunotherapy approaches: IL-6 inhibitors, such as tocilizumab, have demonstrated efficacy in inflammatory conditions, but their role in treating psychiatric comorbidities remains under investigation. Future research should assess their long-term safety and efficacy in neuroinflammation-driven psychiatric disorders [182].

### 6.2. Challenges in Screening and Diagnosis

A fundamental barrier in managing psychiatric and internal disease comorbidities is the symptom overlap between conditions. Fatigue, sleep disturbances, and appetite changes are hallmark features of both depression and chronic internal diseases (e.g., cardiovascular disease, diabetes), complicating accurate diagnosis [183].

Future Directions: The integration of psychiatric scales (e.g., PHQ-9, GAD-7) with inflammatory biomarkers (CRP, IL-6) could improve diagnostic specificity, enabling differentiation between primary psychiatric disorders and inflammation-driven secondary conditions. Additionally, advanced imaging techniques (e.g., functional MRI) may help identify neurobiological patterns associated with specific comorbidities, enhancing diagnostic precision [184].

Multidisciplinary screening protocols that involve both mental health and internal medicine specialists could bridge gaps in diagnostic accuracy. Emerging machine learning algorithms applied to electronic health record (EHR) data may also uncover novel symptom patterns predictive of comorbidities, guiding early intervention [185].

### 6.3. Technological Integration

Limited Utilization of Advanced Monitoring Tools: While telemedicine and wearable devices hold transformative potential for managing comorbidities, their integration into routine care remains limited. Emerging biosensors capable of tracking IL-6 and CRP offer real-time insights into inflammation-driven disease progression, yet these remain largely in the research phase [186].

Barriers to Implementation: The scalability of wearable biosensors is limited by high production costs, data security concerns, and usability challenges, particularly among older adults and individuals with limited technological literacy [185]. Addressing these barriers requires collaboration across biomedical engineering, healthcare policy, and clinical research to develop cost-effective, user-friendly solutions with robust data protection measures [186].

#### Future Outlook and Directions

The integration of wearable biosensors and remote monitoring technologies into routine clinical practice is projected to advance significantly in the coming years. Recent developments in electrochemical impedance spectroscopy-based biosensors have demonstrated feasibility in real-time CRP detection within interstitial fluid, highlighting their potential for continuous inflammation monitoring [185]. Additionally, telemedicine is increasingly recognized as an essential component of cardiovascular and metabolic disease management, as emphasized in the latest European Society of Cardiology (ESC) guidelines [186].

To optimize the implementation of these technologies, future efforts should focus on:

- Validating the clinical accuracy and reliability of biosensors through large-scale, multi-center trials.
- Developing regulatory frameworks that standardize the integration of these tools into clinical practice.
- Improving accessibility by reducing production costs and expanding reimbursement policies for telemedicine and digital health interventions.

Addressing these barriers will be critical in harnessing the full potential of wearable biosensors and telemedicine, ultimately transforming the management of comorbid conditions, particularly in cardiometabolic and inflammatory diseases, and improving long-term patient outcomes worldwide.

## 7. Conclusions

This discussion aligns with the overarching goal of this review—to highlight the central role of neuroinflammation in the comorbidity of psychiatric disorders and internal diseases. By synthesizing current evidence and identifying actionable pathways, it underscores the importance of integrated care models and targeted therapies in addressing these interlinked conditions.

Future efforts must prioritize the development of precision medicine approaches that leverage validated biomarkers, advanced monitoring technologies, and multidisciplinary collaboration. Establishing longitudinal studies and refining diagnostic tools to differentiate overlapping symptoms will be essential in guiding timely and effective interventions. Furthermore, addressing disparities in research and care through inclusive and culturally sensitive strategies can ensure equitable access to emerging innovations.

Moving forward, a unified effort across research, technology, and clinical practice will be critical to mitigating the burden of these complex comorbidities. By fostering partnerships between mental health and internal medicine specialists and embracing patient-centered care, the field can pave the way for transformative advancements in the prevention and management of these interrelated conditions. Key unanswered questions remain regarding how best to integrate these innovations into diverse healthcare systems and how to overcome barriers to their equitable adoption globally.

Addressing these gaps not only has the potential to improve individual patient outcomes but also to reduce the global healthcare burden posed by these interconnected conditions. By prioritizing integrated and innovative approaches, the scientific community can transform the care of patients with comorbid psychiatric and internal diseases, ultimately reducing the global health burden and significantly improving quality of life. This commitment to advancing research and clinical practice must be matched by policies that support equitable healthcare delivery, ensuring that no patient is left behind in the pursuit of improved outcomes.

## Figures and Tables

**Table 1:** Summary of key relationships depicted in Figure 1.

Pathway	Mechanism	References
Neuroinflammation → HPA Axis Dysregulation	Chronic inflammation disrupts cortisol regulation	Mikulska et al., 2021 [7]
Neuroinflammation → ANS Dysfunction	Inflammation increases sympathetic activation	Kenwood et al., 2022 [8]
HPA Axis Dysregulation → Depression	Sustained cortisol elevation damages neural circuits	Herman et al., 2016 [9]
ANS Dysfunction → Anxiety	Autonomic imbalance amplifies stress responses	Kenwood et al., 2022 [8]
Depression → Cardiovascular Disease	Inflammation and autonomic dysfunction increase risk	Vaccarino et al., 2020 [10]
Anxiety → Cardiovascular Disease	Chronic stress and inflammation impair vascular function	Tuomisto et al., 2006 [11]
Depression → Diabetes Mellitus	Inflammatory cytokines worsen insulin resistance	Alzoubi et al., 2018 [12]

**Table 2:** Summary of key relationships depicted in Figure 2.

Pathway	Mechanism	References
IL-6 → Systemic Inflammation	IL-6 promotes chronic systemic inflammation and disrupts immune homeostasis	[30]
CRP → Systemic Inflammation	Elevated CRP levels are associated with increased risk of psychiatric and cardiovascular diseases	[31]
TNF-α → Systemic Inflammation	TNF-α induces pro-inflammatory cascades, exacerbating metabolic and psychiatric conditions	[32]
Systemic Inflammation → Anxiety	Systemic inflammation contributes to hyperactive stress responses and emotional dysregulation	[33]
Systemic Inflammation → Depression	Inflammation alters neurotransmitter systems and promotes depressive symptoms	[34]
Systemic Inflammation → Diabetes Mellitus	Inflammatory cytokines worsen insulin resistance, leading to metabolic dysfunction	[35]
Systemic Inflammation → ANS Dysfunction	Chronic inflammation disrupts autonomic nervous system balance, increasing cardiovascular risk	[36]
Systemic Inflammation → Cardiovascular Disease	Inflammation promotes endothelial dysfunction, increasing CVD risk	[37]
HPA Axis Dysregulation → Depression	Sustained cortisol elevation damages neural circuits, contributing to depression	[38]
ANS Dysfunction → Cardiovascular Disease	Autonomic dysfunction exacerbates vascular dysfunction and increases cardiovascular events	[39]

## Data Availability

The original contributions presented in this study are included in the article. Further inquiries can be directed to the corresponding author.
